# Estimating exposure to neighborhood crime by race and ethnicity for public health research

**DOI:** 10.1186/s12889-021-11057-4

**Published:** 2021-06-05

**Authors:** Evans K. Lodge, Cathrine Hoyo, Carmen M. Gutierrez, Kristen M. Rappazzo, Michael E. Emch, Chantel L. Martin

**Affiliations:** 1 Department of Epidemiology, Gillings School of Global Public Health, University of North Carolina at Chapel Hill, 135 Dauer Drive, Chapel Hill, NC 27599 USA; 2 Carolina Population Center, University of North Carolina at Chapel Hill, Chapel Hill, USA; 3 School of Medicine, University of North Carolina at Chapel Hill, Chapel Hill, USA; 4 Department of Biological Sciences, Center for Human Health and the Environment, North Carolina State University, Chapel Hill, USA; 5 Department of Public Policy, University of North Carolina at Chapel Hill, Chapel Hill, USA; 6 Office of Research and Development, U.S. Environmental Protection Agency, Research Triangle Park, Durham, NC USA; 7 Department of Geography, University of North Carolina at Chapel Hill, Chapel Hill, USA

**Keywords:** Police-reported crime, Race and ethnicity, Crime exposure, Police, Policing

## Abstract

**Background:**

Police-reported crime data (hereafter “crime”) is routinely used as a psychosocial stressor in public health research, yet few studies have jointly examined (a) differences in crime exposure based on participant race and ethnicity, (b) differences in measures of crime exposure, and (c) considerations for how exposure to police is captured in police-recorded crime data. We estimate neighborhood exposure to crime and discuss the implications of structural differences in exposure to crime and police based on race and ethnicity.

**Methods:**

Using GPS coordinates from 1188 participants in the Newborn Epigenetics Study, we estimated gestational exposure to crime provided by the Durham, North Carolina, Police Department within (a) 800 m and (b) the Census block group of residence. We controlled for non-overlapping spatial boundaries in crime, Census, residential, and police data to report crime spatial (crime per km^2^) and population (crime per 1000 people per km^2^) density.

**Results:**

We demonstrate dramatic disparities in exposure to crime based on participant race and ethnicity and highlight variability in these disparities based on the type of crime and crime measurement method chosen.

**Conclusions:**

Public health researchers should give thoughtful consideration when using police-reported crime data to measure and model exposure to crime in the United States, as police-reported data encompasses joint exposure to police and crime in the neighborhood setting.

**Supplementary Information:**

The online version contains supplementary material available at 10.1186/s12889-021-11057-4.

## Background

The current sociopolitical context in the United States raises important questions about the health effects of exposure to crime, police, and policing in the neighborhood environment. Exposure to police-reported crime is a well-documented psychosocial stressor and is associated with poor birth outcomes [1–4], higher perceived stress [5], and poor cardiometabolic [6, 7] and mental health [8, 9]. Such neighborhood-level psychosocial stressors are unequally distributed across the United States, with lower-income and minority populations bearing the intersecting burdens of poverty and structural racism [10] in a manner that increases exposure to crime, police, and physical violence [11, 12]. This is particularly salient for Black and Latinx communities in the US, who are exposed to much higher levels of neighborhood police-reported crime than White populations due to locally- and federally-enforced policies that have a net effect of concentrating and entrenching residential segregation, poverty, unequal educational opportunities, and police surveillance in minority neighborhoods [10, 13, 14]. Researchers often acknowledge the unequal distribution of neighborhood police-reported crime exposure across racial and ethnic groups by conducting single group analyses [15, 16] or stratifying models by race and ethnicity [1–3, 17]. However, the literature is inconsistent in the application of police-reported crime data for epidemiologic research, making inferences across studies difficult.

Several methods are employed in the public health literature to estimate neighborhood crime exposure, including self-reported experiences of victimization [6, 18] and police-reported crime. Police-reported crime measures (hereafter referred to as “crime”) are often aggregated within the Census tract (average population of 2500–8000) [1, 9], Census block group (average population of 600–3000) [2, 5], or other local neighborhood or community area of residence to generate crime counts and rates for public health research [4]. Studies also utilize more participant-centric approaches by capturing crime occurring within a set distance (800 m residential buffer, etc.) of participant residence [19]. Messer et al compared the use of block group crime exposure and crime exposure within an 800 m residential buffer, finding that block group violent crime was associated with adverse birth outcomes, while violent crime counts within 800 m were not [3]. Spatial cluster analysis (“hotspot analysis”) has also been proposed as a useful approach for crime exposure estimation [20], although it is employed less often [8] than Census tract or block group estimation. We also acknowledge examples of crime data analysis at the zip code, metropolitan-statistical area, state, and other levels in the literature [21–23], but we focus here on the use of reported crime for local cohort studies in the public health literature.

As epidemiologic research continues to embrace the use of geographic information systems (GIS) and other analytic tools to evaluate spatial data, their application to geocoded crime data should be critically evaluated. The use of various boundaries to capture neighborhood crime exposure make comparisons between studies difficult, as do different approaches to evaluate crime spatial density versus population density. Measuring “neighborhood” itself is challenging, forcing many researchers to use administrative boundaries provided by the US Census (e.g. block groups) instead of locally-defined neighborhood areas. Many publications also fail to discuss potential measurement error caused by non-overlapping geographic boundaries (ex. a Census tract extending beyond the crime reporting area of the nearest municipal police department), which can be conceived of as a fundamental problem of missing data.

Even if exposure to police-reported crime is perfectly measured (e.g. with no measurement error at the individual level), researchers often fail to discuss what such exposure actually represents. Police-reported crime is *not* solely a measurement of criminal activity. Due to over-policing of neighborhoods with large Black, Latinx, and other minority populations [10, 24, 25], physical proximity to police-reported crime captures some combination of exposure to (a) crime and (b) police. The hypothetical impacts of this mixed crime-police exposure vary based on neighborhood composition due to racially and ethnically disparate experiences of police surveillance and violence [26–28]. As an example, the US “War on Drugs” policies of the 1970s–80s resulted in disproportionate exposure to punitive policing and police violence among Black Americans despite data that demonstrate equivalent rates of drug offenses regardless of race and ethnicity [24, 25]. As such, police-reported data on drug offenses systematically misses drug offenses in majority-White neighborhoods while capturing a mixture of exposure to drug offenses *and* exposure to police surveillance in majority-Black neighborhoods.

Using data from the Newborn Epigenetics Study (NEST) and the Durham, North Carolina, Police Department (DPD), we estimated participant exposure to violent crime, drug-related crime, and burglary during the gestational period to demonstrate various approaches and challenges that researchers should consider when estimating reported crime exposure. We leveraged participant residential GPS coordinates and geocoded crime data from the DPD to estimate gestational exposure to crime (a) within 800 m of participant residence and (b) within the Census block group of participant residence, accounting for potential measurement error due to non-overlapping residential, Census, and crime data boundaries. We estimated spatial crime density (crimes per km^2^) and population crime density (crimes per 1000 people per km^2^) to demonstrate large disparities in gestational crime exposure based on participant self-reported race and ethnicity, providing hypothetical examples where different measurements of crime density may be more or less appropriate. We also demonstrate the importance of evaluating seasonal and spatial variation in crime exposure. Finally, we discuss the importance of evaluating police-reported crime data as data regarding both criminal activity *and* police presence in the neighborhood environment. Although we do not disentangle the mixed effects of crime and policing here, we provide a number of considerations and general advice for researchers interested in using these data in the future.

## Methods

### Study population

Participant data was provided by the Newborn Epigenetics Study (NEST), a birth cohort of 2681 parent-child pairs enrolled from 2005 to 2011 at Duke University Hospital and Durham Regional Hospital Obstetrics in Durham, North Carolina, with follow-up into early childhood. The catchment area for these hospital systems combines the central North Carolina counties of Orange, Durham, and Wake, as well as individuals living in Chapel Hill, Durham, Raleigh, and the surrounding area [29]. Participants had to be ≥18 years of age, pregnant, and English-speaking at enrollment. Among eligible participants, individuals who did not intend to retain custody of their child, intended to move in the next 3 years, or had an established HIV infection were excluded from participation. Participant sociodemographic characteristics were collected by trained interviewers at enrollment and included self-reported participant age, race, ethnicity, educational attainment, and residential address. Race and ethnicity were recorded as “African American/Black” (referred to as “Black” throughout this manuscript), “Hispanic” (referred to as “Latinx” throughout this manuscript), and “White.” Pregnancy data and birth outcomes were collected by medical abstraction immediately following birth. The NEST sampling frame and study design have been previously described in detail [29, 30]. We excluded all NEST participants who did not provide an address at enrollment (*n* = 273), provided an address outside of Durham city limits (*n* = 820), experienced any portion of their gestation outside of the period of available crime data (*n* = 252), had a non-singleton birth (*n* = 75), and did not self-identify as Black, Latinx, or White or were missing data on race/ethnicity (*n* = 73). A total of 1188 participants in 149 unique block groups within the City of Durham were eligible for our analysis. Study protocol and design were approved by the Duke University and University of North Carolina at Chapel Hill Institutional Review Boards.

### Durham police department police-reported crime data

The Durham Police Department (DPD) provided police-reported crime data for the City of Durham between April 1, 2006, and December 31, 2012. Each crime event was reported with a date, description, and donut-masked GPS coordinate within the city limits. Donut masking shifts GPS locations in a random direction within a pre-specified minimum and maximum distance to protect against reverse geocoding and ensure anonymity while introducing only minor random error in the associated data [31]. DPD crime categories were based on Federal Bureau of Investigations Uniform Crime Reporting standards [32]. Examples include “aggravated assault,” “burglary – forcible entry,” and “larceny – pocket picking.”

For this analysis, we focused on reports of violent crime, drug-related crime, and burglary. These do not represent all types of crime that could be important for public health and serve only as a sample of different crime classifications that we hypothesized would be variably distributed across the city of Durham. Crimes categorized as “violent crime” included reports of homicide, rape, sexual assault, aggravated assault, simple assault, and robbery. We also created a separate “violent crime” variable that excluded reports of rape and sexual assault as a sensitivity analysis due to concerns regarding inaccuracies and underreporting of sexual violence [33]. Crimes categorized as “drug-related crime” included reports of transporting, selling, buying, possessing, or using controlled substances, the majority of which are drug/narcotic violations although a very small minority of reports may be related to inappropriate transportation, selling, etc. of alcohol. The “drug-related crime” label does not include driving while impaired or any reports filed under “liquor law” violations. “Burglary” was reported and recorded as its own category by the DPD.

### Crime exposure assessment

Participant residential addresses were collected at study enrollment, geocoded by Duke University staff, and used to create gestational crime exposure variables for each crime type (“violent,” “drug-related,” and “burglary”). We first counted reported crimes of each type occurring within 800 m of participant residence on or after the last menstrual period and up to the date of delivery. Given that crime data is only available within the city of Durham, this approach is subject to potential measurement error for individuals living within 800 m of the city limits. To control for this potential misclassification, we divided each participants’ 800 m crime count by the area of their 800 m buffer contained entirely within the city limits (Eq. 1).

1 $$ \frac{800m\  crime\ count}{Area\left(800m\  buffer\ within\ city\ limits\right)} $$

We used a similar approach to measure gestational crime exposure occurring within the Census block group of participant residence, dividing a count of all crimes within the block group by the area of the block group contained within Durham city limits (Eq. 2). As such, spatial crime density from Eqs. 1 and 2 is reported as crimes per km^2^.

2 $$ \frac{block\ group\ crime\ count}{Area\left( block\ group\ within\ city\ limits\right)} $$

We also calculated crime rates corrected for population density using 2006–2010 American Community Survey (ACS) data provided by IPUMS [34]. We estimated the population within 800 m of each participant residential address by (a) calculating the proportion of the area of each Census block group within 800 m of participant residence *and* within Durham city limits, (b) multiplying this proportion by the same block group’s population, and (c) summing each of these estimated populations (Eq. 3).

3 $$ \sum \limits_{i=1}^n\left[\left(\frac{Area\left( block\ {group}_i\  within\ 800m\  buffer\ and\ city\ limits\right)}{Area\left( block\ {group}_i\right)}\right)\times Population\left( block\ {group}_i\right)\right] $$

We estimated the population of each Census block group by multiplying the proportion of the block group contained within Durham city limits by the same block group’s 2006–2010 ACS population estimate (Eq. 4).

4 $$ \left(\frac{Area\left( block\ group\ within\kern0.5em city\ limits\right)}{Area\left( block\ group\right)}\right)\times Population\left( block\ group\right) $$

We divided the estimated crimes per km^2^ from Eq. 1 and Eq. 2 by the estimated populations from Eq. 3 and Eq. 4, respectively, to capture crime density within the population as crimes per 1000 people per km^2^.

**Fig. 1:**
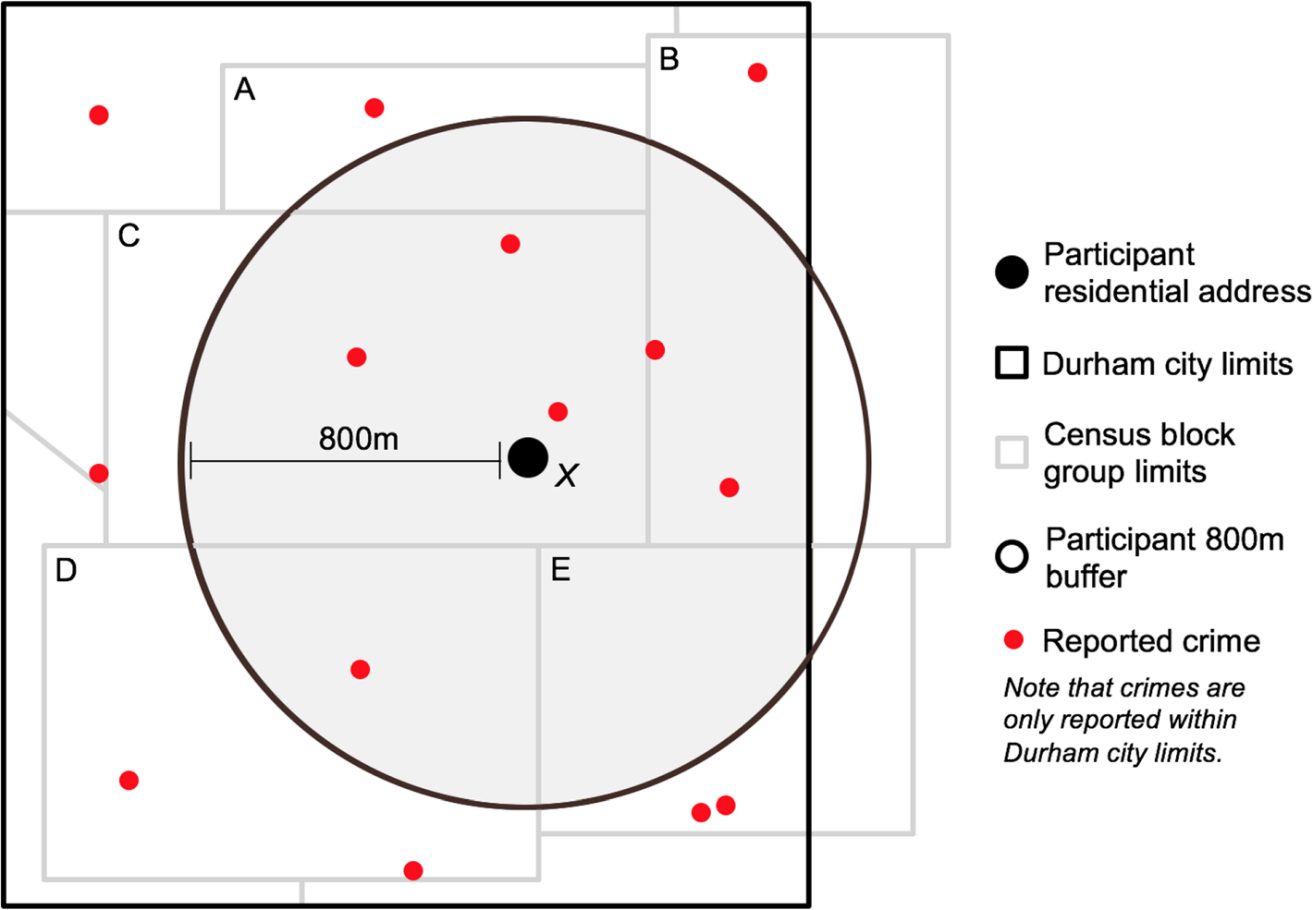
To estimate area-level gestational crime exposure for participant *X*: **(1)** Count all reported crimes within 800 m that occurred between *X*’s last menstrual period and date of delivery. **(2)** Calculate the area of *X*’s 800 m buffer within Durham city limits (shaded portion of circle). For participants living ≥800 m from city limits, this is equal to *π*(800*m*)^2^. For *X*, this is equal to [*π*(800*m*)^2^ − *Area*(*unshaded portion of circle*)]. **(3)** Estimate the population within *X*’s 800 m buffer contained within Durham city limits (shaded portion of circle). For block group A, this is equal to: $\left[\left(\frac{Area\left( shaded\ portion\ of\ A\right)}{Area(A)}\right)\ast Population(A)\right]$. Repeat this for block groups B-E, and sum these populations. **(4)** Dividing the crime count from [1] by the area in [2] results in a measure of crimes per km^2^. **(5)** Dividing the crimes per km^2^ derived in [4] by the population estimated in [3] results in a measure of crimes per 1000 people per km^2^ To estimate block group level gestational crime exposure for *X*: **(1)** Count all reported crimes within the block group of residence, C, that occurred between *X*’s last menstrual period and date of delivery. **(2)** Calculate the proportion of C’s area within Durham city limits (1, in this example). **(3)** Multiply the area of C by the proportion derived in [2]*.* **(4)** Multiply the population of C by the proportion derived in [2]*.* **(5)** Dividing the crime count from [1] by the area in [3] results in a measure of crimes per km^2^. **(6)** Dividing the crimes per km^2^ derived in [5] by the population estimated in [4] results in a measure of crimes per 1000 people per km^2^.

Thus, we have four measurements of gestational crime exposure for each type of crime (“violent,” “drug-related,” and “burglary”): (a) crime within an 800 m residential buffer per km^2^, (b) crime within an 800 m residential buffer per 1000 people per km^2^, (3) crime within the Census block group of residence per km^2^, and (4) crime within the Census block group of residence per 1000 people per km^2^. We refer to crime within 800 m of participant residence as “area-level crime,” and crime within the Census block group of participant residence as “block group crime.” Fig. 1 provides a hypothetical worked example of these exposure estimation methods. We also estimated crime per km^2^ and crime per 1000 people per km^2^ with residential buffer sizes of 400 m and 1600 m using the same methods outlined above.

### Statistical analysis

We summarized reported crime and participant sociodemographic characteristics using counts and proportions for categorical variables and means and standard deviations for continuous variables. We quantified seasonal variation in crime rates by counting the number of each type of crime within each month of the study period, and visualized geographic concentration of reported crime by plotting their kernel densities within Durham city limits [35]. We used violin plots [36] and boxplots of the 25th quartile, median, 75th quartile, and Tukey whiskers [37] to inspect disparities in crime exposure based on participant race and ethnicity. We also performed several sensitivity analyses to examine the robustness of our estimates to crime categorization (excluding reported rape and sexual assault from violent crime category) and varying buffer size (400 m and 1600 m). All data organization, visualization, and statistical analysis was conducted in *R* (version 3.5.3) [38].

## Results

A total of 167,162 crimes were reported by the DPD between April, 2006, and December, 2012 (Table 1). The most common DPD reported crime types were larceny (28.6%), burglary (14.3%), and vandalism (9.6%). We also created two new categories for this analysis: violent crime (13.0%, including simple and aggravated assault, homicide, rape, robbery, and sexual assault) and drug-related crime (6.3%, including drug equipment/paraphernalia charges and drug/narcotic violations).

**Table 1:** Characteristics of crime data provided by the Durham Police Department for the period between April 1, 2006, and December 31, 2012. We use “Violent Crime,” “Drug-Related Crime,” and “Burglary” as major categories of crime for this project. “Other Crime” refers to all crimes not included in our analysis. Each crime type is calculated as a count (proportion). Column percentages may not sum to 100% due to rounding

Total Reported Crimes	167,162
**Violent Crime**	**21,658 (13.0%)**
Aggravated Assault	4721 (2.8%)
Homicide	147 (0.1%)
Rape	497 (0.3%)
Robbery	5154 (3.1%)
Sexual Assault	814 (0.5%)
Simple Assault	10,325 (6.2%)
**Drug-Related Crime**	**10,552 (6.3%)**
Drug Equipment/Paraphernalia	1196 (0.7%)
Drug/Narcotic Violations	9356 (5.6%)
**Burglary**	**23,976 (14.3%)**
**Other Crime**	**110,976 (66.4%)**

Sociodemographic characteristics of participants included in this study are displayed in Table 2. The average participant in our analytic sample was 27.8 years old at delivery, and the majority of participants in our analysis were Black (53%). There were striking disparities in educational attainment based on participant race and ethnicity, with 60% of self-identified Latinx participants reporting less than a high school education, compared to 20% of Black participants and only 7% of White participants. Children born to Black participants were born earlier (38.4 weeks) and lighter (3072 g), on average, than children born to Latinx (39.0 weeks, 3318 g) and White (39.3 weeks, 3382 g) participants.

**Table 2:** Sociodemographic characteristics of participants in this analysis (*n* = 1188) from the Newborn Epigenetics Study (NEST). Continuous variables are displayed as mean (standard deviation, SD) and categorical variables are displayed as count (percentage). Column percentages may not sum to 100% due to rounding

	All Eligible Participants (***n*** = 1188)	Black (***n*** = 627)	Latinx (***n*** = 308)	White (***n*** = 253)
**Participant Age at Delivery**				
Mean (SD)	27.8 (± 5.8)	26.4 (± 5.8)	28.1 (± 5.7)	30.7 (± 4.9)
Missing	1 (0.1%)	1 (0.2%)	0 (0%)	0 (0%)
**Participant Race/Ethnicity**				
Black	627 (53%)	–	–	–
Latinx	308 (26%)	–	–	–
White	253 (21%)	–	–	–
**Participant Educational Attainment**				
< High School	329 (28%)	127 (20%)	184 (60%)	18 (7%)
High School Grad	279 (23%)	196 (31%)	62 (20%)	21 (8%)
Some College	223 (19%)	175 (28%)	24 (8%)	24 (9%)
College Grad	293 (25%)	98 (16%)	13 (4%)	182 (72%)
Missing	64 (5.4%)	31 (4.9%)	25 (8.1%)	8 (3.2%)
**Gestational Age (Weeks)**				
Mean (SD)	38.8 (± 2.4)	38.4 (± 2.6)	39.0 (± 2.3)	39.3 (± 1.9)
**Delivery Weight (grams)**				
Mean (SD)	3201 (± 606.5)	3072 (± 625.1)	3318 (± 556.3)	3382 (± 541.7)
Missing	15 (1.3%)	7 (1.1%)	4 (1.3%)	4 (1.6%)

**Fig. 2:**
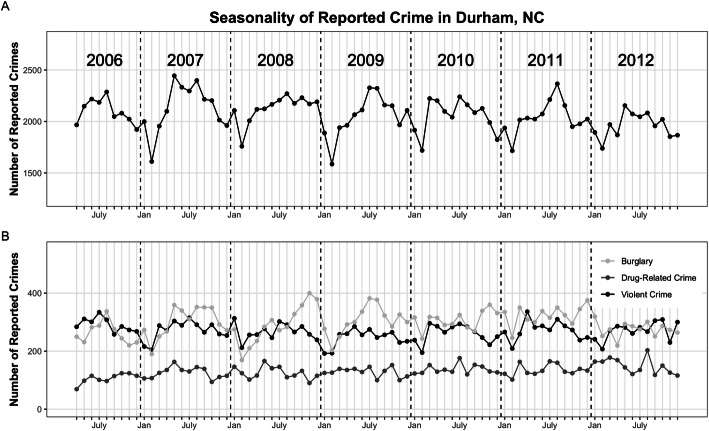
Seasonal variation in **A** total police-reported crime provided by the Durham Police Department and **B** violent crime, drug-related crime, and burglary used for this analysis. Each point refers to the crime count reported during one month (April, 2006 – December, 2012). Total reported crime peaks in the summer and decreases during the winter. Violent crime, drug-related crime, and burglary show a similar seasonal pattern with less overall variation

Total reported crime (*n* = 167,162) showed strong seasonal variation (Fig. 2A), with peaks in the summer months, troughs in the winter, and no obvious secular trend towards more or less crime over the study period. This seasonal patterning of crime is consistent with what is observed in the criminology and public health literature [39]. The violent crime, drug-related crime, and burglaries used in our analysis showed a similar seasonal pattern with less obvious peaks and troughs (Fig. 2B).

**Fig. 3:**
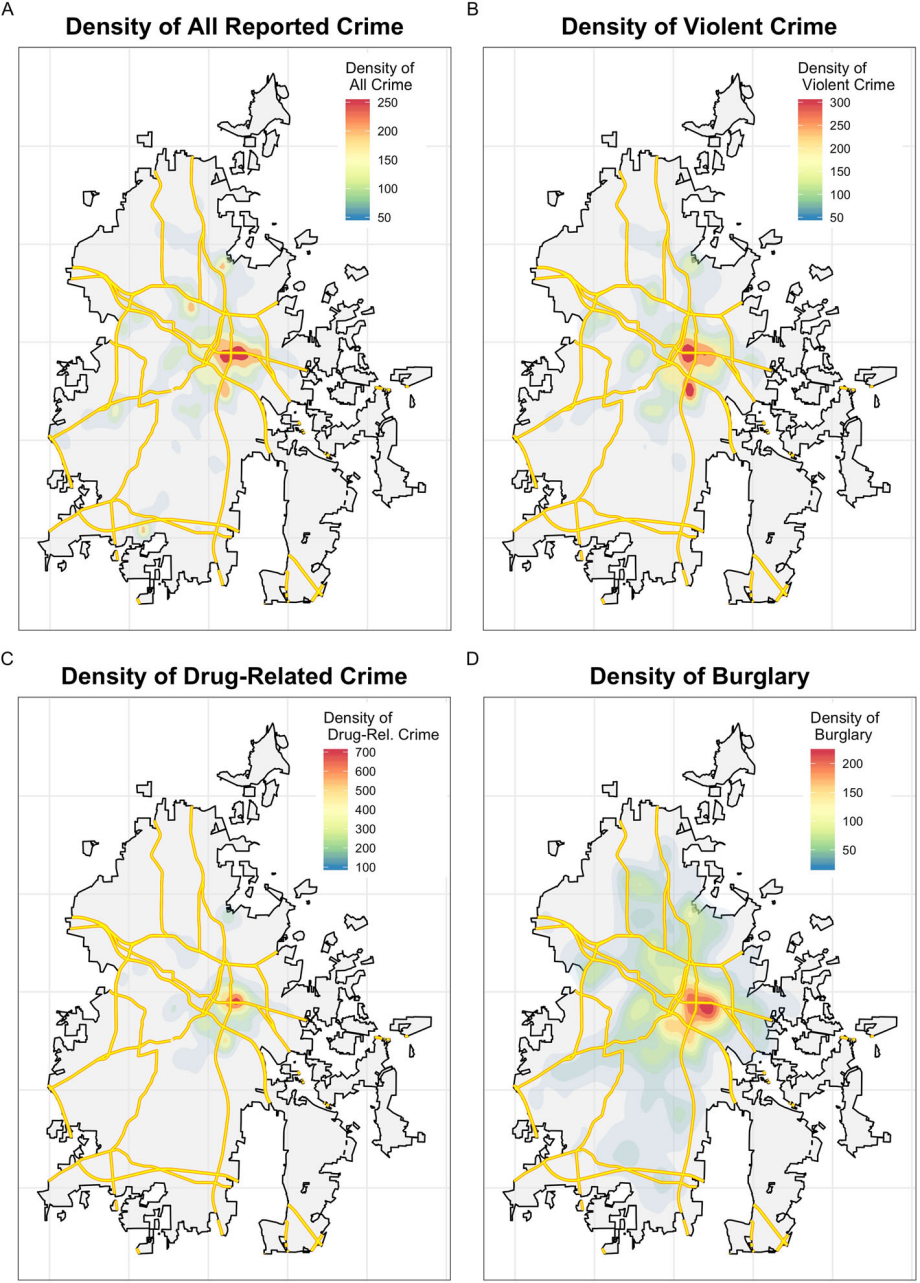
Kernel probability density of **A** all reported crime, **B** violent crime, **C** drug-related crime, and (**D**) burglaries between 2006 and 2012. Every type of crime is concentrated in the Durham city center area. There were no apparent temporal changes in the spatial distribution of each crime (not shown). Primary and secondary roads are displayed in yellow, and Durham city limits are displayed in black. Maps and image created by E.K. Lodge

Crime reports cluster strongly in the city center area for all 167,162 reported crimes (Fig. 3A), violent crime (Fig. 3B), drug-related crimes (Fig. 3C), and burglaries (Fig. 3D). Crime probability densities demonstrate particularly high concentration of drug-related crime reports in the city center area, while reported burglaries are the least concentrated. There were no differences in the spatial distribution of each type of crime based on the year of crime report over the study period.

**Fig. 4:**
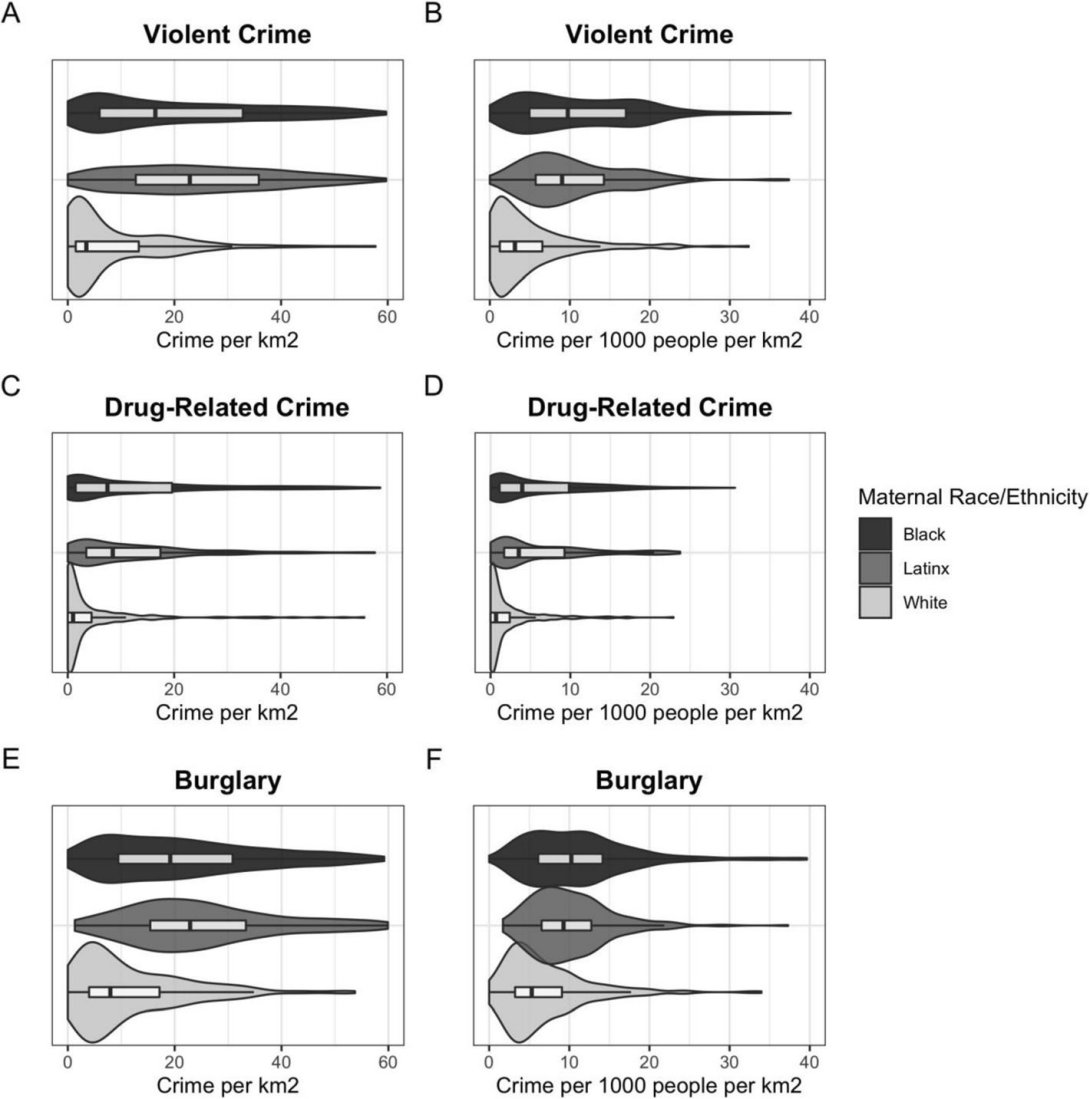
Frequency of **A**, **B** violent crime, **C**, **D** drug-related crime, and **E**, **F** burglary exposure within 800 m of participant residence during gestation based on self-reported race and ethnicity. Plots on the left **A**, **C**, and **E** display crimes per km^2^, and plots on the right **B**, **D**, and **F** display crimes per 1000 people per km^2^. Violin plots display the smoothed kernel density of crime, while boxplots display the 25th quartile, median, 75th quartile, and Tukey whiskers (outliers are not displayed for simplicity). Black and Latinx participants are exposed to much higher levels of crime during gestation than white participants

Figure 4 displays the frequency of each type of crime within 800 m of participant residence, with plots on the left (*4A*, *4C*, *4E*) displaying crimes per km^2^ and plots on the right (*4B*, *4D*, *4F*) displaying crimes per 1000 people per km^2^. Gestational exposure to crime is strongly patterned by participant race/ethnicity, with self-identified Black and Latinx participants exposed to higher area-level crime than White participants regardless of type (violent, drug-related, and burglary) and method of density estimation (crimes per km^2^ or per 1000 people per km^2^). As an example, the median White participant was exposed to only 3.6 (IQR: 1.5, 14.4) violent crimes per km^2^ within 800 m of residence during gestation, compared to 23.9 (IQR: 13.5, 37.3) for the median Latinx participant and 19.4 (IQR: 7.0, 38.8) for the median Black participant (*4A*). Adjusting for population density (*4B*) slightly lessens this disparity, although White participants were still exposed to roughly 25% the number of violent crimes per 1000 people per km^2^ as Black and Latinx participants. Disparities in exposure to reported drug-related crime (*4C*, *4D*) within 800 m were even larger than those identified for violent crime, with White participants experiencing approximately 10% of the reported drug-related crimes as Black and Latinx participants when measured as crimes per km^2^, and approximately 15% when measured as crimes per 1000 people per km^2^. Reported burglary (*4E*, *4F*) within 800 m of participant residence showed the least variation by participant race and ethnicity, although White participants were still exposed to between 30 and 50% of the burglaries as Black and Latinx participants. Uncorrected crime counts (raw counts of crime within 800 m of residence or within the Census block group of residence) exaggerate these disparities, as White participants are more likely to live within 800 m of the Durham city limits or in a block group that extends beyond the city limits.

Measuring crime density within the block group of participant residence also yielded striking disparities, with the median White participant again experiencing approximately 25% of the violent crime exposure, 20% of the drug-related crime exposure, and 40% of the burglary exposure as the median Black or Latinx participant (Supplemental Figure 1).

Sensitivity analyses of violent crime excluding rape and sexual assault did not result in any noteworthy changes in the seasonality, spatial density, or exposure disparities of violent crime due to the extremely small number of reported events of rape and sexual assault. Sensitivity analyses using smaller (400 m) residential buffers led to increased disparities by race and ethnicity, with the median White participant exposed to approximately 15% of the violent crime exposure and 30% of the burglary exposure as the median Black or Latinx participant; notably, the median White participant was exposed to zero drug-related crime reports when exposure was estimated using 400 m buffers, highlighting the racialized concentration of drug-related policing in minority neighborhoods (Supplemental Figure 2). Crime exposure using larger (1600 m) residential buffers led to decreased disparities by race and ethnicity, with the median White participant exposed to approximately 40% of the violent crime exposure, 20% of the drug-related crime exposure, and 60% of the burglary exposure as the median Black or Latinx participant (Supplemental Figure 3).

## Discussion

In this study, we summarize four potential measurements of neighborhood police-reported crime exposure using geocoded crime data, drawing attention to large disparities in crime exposure based on race and ethnicity and highlighting important considerations associated with seasonal and spatial variation in reported crime levels. We estimated gestational crime exposure to demonstrate these approaches with regard to a sensitive period of human development that is directly tied to multiple health outcomes, but note that exposure could be estimated within any period of interest to public health researchers. Taken together, our findings emphasize several key challenges that epidemiologists and other public health researchers should consider when incorporating geospatial police-reported crime exposure data into work regarding the neighborhood environment.

First, our study demonstrates pronounced disparities in neighborhood crime exposure, with Black and Latinx residents of Durham exposed to systematically higher police-reported crime than White residents. Due to centuries of entrenched racial residential segregation [13] and structural racism that continue to shape Black and Latinx neighborhoods, racial and ethnic minority communities experience higher concentrations of poverty, unemployment, police surveillance, and numerous other social stressors [10] that contribute to disparate police-reported crime exposure. This has immediate implications for researchers who choose to model the effect of crime exposure on a health outcome while including terms for race and ethnicity in their statistical models, as they will smooth over data that fundamentally does not exist (ex. White residents with very high police-reported neighborhood crime) and generate estimates of effect that do not correspond to any real-world police-reported crime exposure [40]. To overcome this issue, researchers should consider utilizing stratified models that incorporate crime data based on participant race and ethnicity.

Model stratification by race and ethnicity, while addressing violations of positivity caused by the outsize burden of police-reported crime exposure among Black and Latinx Americans, adds an additional layer of complexity to public health research utilizing reported crime data: the question of what exposure, exactly, police-reported crime actually measures. While police-reported crime may be statistically correlated with true rates of crime (particularly for more serious violent offenses such as robbery, aggravated assault, and homicide) [41], it is also, by definition, highly correlated with police presence in a neighborhood or area. Due to the long and ongoing history of punitive policing in Black, Latinx, and other minority communities in the United States [25], police-reported crime exposure represents a distinct set of psychosocial and physical stressors in a neighborhood based on the neighborhood’s ethnoracial composition. For Black, Latinx, and other over-policed populations in the United States, police-reported crime data captures exposure to crime *and* exposure to police in the neighborhood; both bring with them the potential for stress, violence, and even death [22, 26–28, 42, 43]. Schwartz and Jahn recently found that the incidence of fatal police violence against Black residents in the Durham-Chapel Hill metropolitan-statistical area (MSA) was 3.58 (95% CI: 0.82, 15.68) times the incidence of fatal police violence against White residents [21]. For Black and Latinx NEST participants living in the Durham-Chapel Hill MSA, fear of police violence is not an abstraction.

Furthermore, White people in the United States are less likely to be reported, charged, or prosecuted for criminal behavior than any other group in the United States [24], meaning that reported crime in majority White residential areas may represent an undercount of actual criminal behavior. White people in the United States are also far less likely to suffer violence or death at the hands of police than Black or Latinx people [26, 27, 44], and are more likely to support increased funding for municipal police departments [45]. Thus, while epidemiologic research on the effect of police-reported crime in majority-White neighborhoods may be a reasonable estimate of stress caused by actual criminal activity in the absence of stress caused by interactions with police, the same research in majority Black or Latinx neighborhoods will capture the layered effect of exposure to crime *and* exposure to potential police violence. These are fundamentally non-comparable exposures, and are impossible to disentangle with police-reported crime data alone. Ideally, future studies should include participant-reported crime victimization data alongside participant-reported data on interactions with the police in order to investigate simultaneous exposure to crime and policing in minority communities [46]. The inclusion of citizen complaints regarding police behavior may also be a useful control to capture policing rather than crime. Unfortunately, these data were not available in NEST.

Second, we demonstrate two methods to measure police-reported crime exposure in the residential area (crime within 800 m of residence and crime within the block group of residence), and two methods to estimate crime density (crime per km^2^ and crime per 1000 people per km^2^). Both methods have been utilized in the epidemiologic literature to date, although estimated crime counts or rates within the Census tract or block group of residence appear to be more common. Both methods are also prone to error if researchers do not explicitly acknowledge, and attempt to control for, the effects of non-overlapping boundaries of crime data, Census tracts or block groups, and residential buffers. As demonstrated, when counting crime that occurred within the Census tract or block group of residence, crime counts or rates will be underestimated for participants in a Census tract or block group that extends beyond the city limits due to missing crime data outside of the city (or any other municipal boundary with police or sheriff recorded crime data). The same “edge effects” or “boundary problems” [47] will occur when using a residential buffer to estimate area-level crime exposure, as any participant whose residential buffer extends beyond the limits of the available crime data will have falsely low reported crime exposure. This finding held true in our data, leading to exaggerated disparities in uncontrolled crime count exposures because White participants were more likely to live near the Durham city limits than Black and Latinx participants (as an example, 29.2% of White participants in our sample lived within 800 m of the Durham city limits, versus 27.3% of Black participants and only 14.0% of Latinx participants). To control for this bias, we recommend that researchers calculate the area of the Census tract, block group, or residential buffer that overlaps with the available crime data, and divide crime counts by this area to generate a measurement of spatial crime density in the neighborhood environment (crime per km^2^). We also demonstrate how to apply similar methods to estimate the population that overlaps with the available crime data to generate a population crime density estimate (crime per 1000 people per km^2^).

The choice between these two crime density metrics (crime per km^2^ versus crime per 1000 people per km^2^) should be made based on the hypothetical mechanism through which reported crime exposure is expected to impact health, keeping in mind that neighborhood racial and ethnic composition will modulate these effects based on experiences of police violence. As a hypothetical example, reported homicide may be so stressful that participants experience a psychosocial and physiological fear response regardless of the surrounding population density. In this context, crime per km^2^ may be most appropriate, as participants experience a dose-response stress effect with increasing homicide exposure that is not buffered by a larger population in the surrounding area. For less physically dangerous or stressful crime, such as larceny, researchers may hypothesize that participants’ stress response is modified by the population density in such a way that participants feel more secure in busier or more populated areas. In this context, crime per 1000 people per km^2^ would be most appropriate. As has been done before, researchers may choose to compare these approaches head-to-head [3]. We provide these hypothetical examples to encourage public health researchers to critically engage with police-reported crime data, acknowledge how different crime density metrics may be more or less fit for particular models of crime exposure, and evaluate distinct mechanisms of effect for distinct types of crime. Hypothetical mechanisms must also account for different experiences of punitive policing and police violence in the population.

While information on the perception of neighborhood crime was not available for all participants in our study, we also recognize the relevance of using distinctly defined, community-derived neighborhood boundaries as opposed to arbitrary spaces derived by Census block groups and buffers. Such “place-based” analyses [48] are context-specific and require community knowledge to conduct, but may provide additional insight into the effects of crime density beyond those derived from larger epidemiologic studies. The estimation methods outlined here can be used with any set of geographic boundaries, and we encourage researchers to use locally-derived and -recognized neighborhood boundaries when appropriate for their research question.

Lastly, aside from a critical evaluation of different methods to estimate crime density and exposure, we also highlight strong seasonal variation in crime that public health researchers should consider. Depending on the time of year, duration of a cross-sectional or longitudinal cohort study, and the type of crime under investigation, seasonal variation in reported crime may cause researchers to miss peak periods of crime exposure and weaken their ability to infer the effect of crime on population health. This seasonal variation, combined with the concentration of crime and policing in the city center [49], may also induce problems related to structural confounding [40], or structural violations of positivity [50].

The estimated disparities in reported crime exposure based on participant race and ethnicity that we present are not without limitations. First, we are currently unable to offer concrete methodological steps for public health researchers interested in disentangling police exposure from crime exposure using only police-reported crime data. We hope that acknowledging the limitations of police-reported data as a marker of actual crime will encourage other researchers to investigate how to better model the health effects of crime and policing as separate exposures. Second, of the 2681 parent-child pairs in NEST, 1493 pairs (55.7%) were excluded. While exclusion based on participant residence outside of the city of Durham (*n* = 820), gestation outside of the temporal availability of crime data (*n* = 252), and non-singleton birth (*n* = 75) would not be expected to bias the representativeness of our analysis regarding gestational exposure to crime in the city of Durham, exclusion based on missing data regarding race/ethnicity (*n* = 73) and residential address (*n* = 273) may cause unpredictable bias in our results by creating a non-representative sample of retained participants. Third, the use of donut masking by the Durham Police Department means that any given crime report during our study period may have been shifted slightly in space. This randomly introduced error would be expected to cause non-differential bias in our findings, but we are unable to access unmasked data to analyze the impacts of donut masking in our sample. Fourth, we would ideally have access to both racial and ethnic data for NEST participants (i.e. non-Latinx Black, Latinx Black, non-Latinx White, etc.), but were limited to categories of “Black,” “Latinx,” and “White” in the provided data. Collapsing racial and ethnic data into one ethnoracial construct smooths over salient health differences at the population level [51], as the “Latinx” label available in NEST almost certainly combines individuals who are racialized as Black, White, and more [52]. This is further complicated in NEST due to the requirement that participants spoke English at enrollment. We encourage epidemiologic researchers collecting data on neighborhood crime exposure (or any other socioenvironmental exposure) to collect and incorporate data on both race and ethnicity (as well as sexuality, gender identity, etc), as individuals occupying multiple marginalized identities [53] may face the highest levels of interpersonal and neighborhood crime and violence.

## Conclusions

A diverse set of methods to estimate individual-level residential exposure to police-reported crime have been used in the public health literature to date, but the modeling implications of different methods are often ignored. Here, we present multiple methods to estimate crime exposure, demonstrate how different methods modulate estimated racial and ethnic disparities in exposure, discuss how these estimation procedures fit with different modeling strategies in public health research, and critically examine the assumption that police-reported crime is a true measure of crime. We also demonstrate that ignoring non-overlapping spatial boundaries in crime, Census, and other geographic data may lead to the overestimation of racial and ethnic disparities in crime exposure in urban areas because White Americans are more likely to live near the geographic edges of their cities of residence than other racial and ethnic groups. Further methodological work to disentangle the mixed health effects of crime and policing, particularly in over-policed neighborhoods in the United States, is critical as efforts to limit exposure to crime and police violence continue.

## Supplementary Information

**Additional file 1: Supplemental Figure 1.** Frequency of (**A**, **B**) violent crime, (**C**, **D**) drug-related crime, and (**E**, **F**) burglary exposure within the Census block group of participant residence during gestation based on self-reported race and ethnicity. Plots on the left (**A**, **C**, and **E**) display crimes per km^2^, and plots on the right (**B**, **D**, and **F**) display crimes per 1000 people per km^2^. Violin plots display the smoothed kernel density of crime, while boxplots display the 25th quartile, median, 75th quartile, and Tukey whiskers (outliers are not displayed for simplicity). **Supplemental Figure 2.** Frequency of (**A**, **B**) violent crime, (**C**, **D**) drug-related crime, and (**E**, **F**) burglary exposure within 400 m of participant residence during gestation based on self-reported race and ethnicity. Plots on the left (**A**, **C**, and **E**) display crimes per km^2^, and plots on the right (**B**, **D**, and **F**) display crimes per 1000 people per km^2^. Violin plots display the smoothed kernel density of crime, while boxplots display the 25th quartile, median, 75th quartile, and Tukey whiskers (outliers are not displayed for simplicity). **Supplemental Figure 3.** Frequency of (**A**, **B**) violent crime, (**C**, **D**) drug-related crime, and (**E**, **F**) burglary exposure within 1600 m of participant residence during gestation based on self-reported race and ethnicity. Plots on the left (**A**, **C**, and **E**) display crimes per km^2^, and plots on the right (**B**, **D**, and **F**) display crimes per 1000 people per km^2^. Violin plots display the smoothed kernel density of crime, while boxplots display the 25th quartile, median, 75th quartile, and Tukey whiskers (outliers are not displayed for simplicity).

## Data Availability

The datasets generated and analyzed during the current study are not publicly available due to the presence of identifiable information on NEST participants, but can be made available from Chantel L. Martin and Cathrine Hoyo (project Principal Investigators) upon reasonable request.

## Abbreviations

NEST: Newborn Epigenetics Study
DPD: Durham Police Department (Durham, North Carolina)
MSA: Metropolitan Statistical Area
