# Supplementary material for: Estimating exposure to neighborhood crime by race and ethnicity for public health research
Source: BMC Public Health. 2021 Jun 5;21:1078. doi: 10.1186/s12889-021-11057-4 (PMC8183080; doi:10.1186/s12889-021-11057-4)
Supplement: Supplementary file 1 — Additional file 1: Supplemental Figure 1. Frequency of (A, B) violent crime, (C, D) drug-related crime, and (E, F) burglary exposure within the Census block group of participant residence during gestation based on self-reported race and ethnicity. Plots on the left (A, C, and E) display crimes per km2, and plots on the right (B, D, and F) display crimes per 1000 people per km2. Violin plots display the smoothed kernel density of crime, while boxplots display the 25th quartile, median, 75th quartile, and Tukey whiskers (outliers are not displayed for simplicity). Supplemental Figure 2. Frequency of (A, B) violent crime, (C, D) drug-related crime, and (E, F) burglary exposure within 400 m of participant residence during gestation based on self-reported race and ethnicity. Plots on the left (A, C, and E) display crimes per km2, and plots on the right (B, D, and F) display crimes per 1000 people per km2. Violin plots display the smoothed kernel density of crime, while boxplots display the 25th quartile, median, 75th quartile, and Tukey whiskers (outliers are not displayed for simplicity). Supplemental Figure 3. Frequency of (A, B) violent crime, (C, D) drug-related crime, and (E, F) burglary exposure within 1600 m of participant residence during gestation based on self-reported race and ethnicity. Plots on the left (A, C, and E) display crimes per km2, and plots on the right (B, D, and F) display crimes per 1000 people per km2. Violin plots display the smoothed kernel density of crime, while boxplots display the 25th quartile, median, 75th quartile, and Tukey whiskers (outliers are not displayed for simplicity). [file 12889_2021_11057_MOESM1_ESM.docx]

**SUPPLEMENTAL IMAGES:**

**Supplemental Figure 1:** Frequency of (**A**, **B**) violent crime, (**C**, **D**) drug-related crime, and (**E**, **F**) burglary exposure within the Census block group of participant residence during gestation based on self-reported race and ethnicity. Plots on the left (**A**, **C**, and **E**) display crimes per km^2^, and plots on the right (**B**, **D**, and **F**) display crimes per 1000 people per km^2^. Violin plots display the smoothed kernel density of crime, while boxplots display the 25^th^ quartile, median, 75^th^ quartile, and Tukey whiskers (outliers are not displayed for simplicity).

**
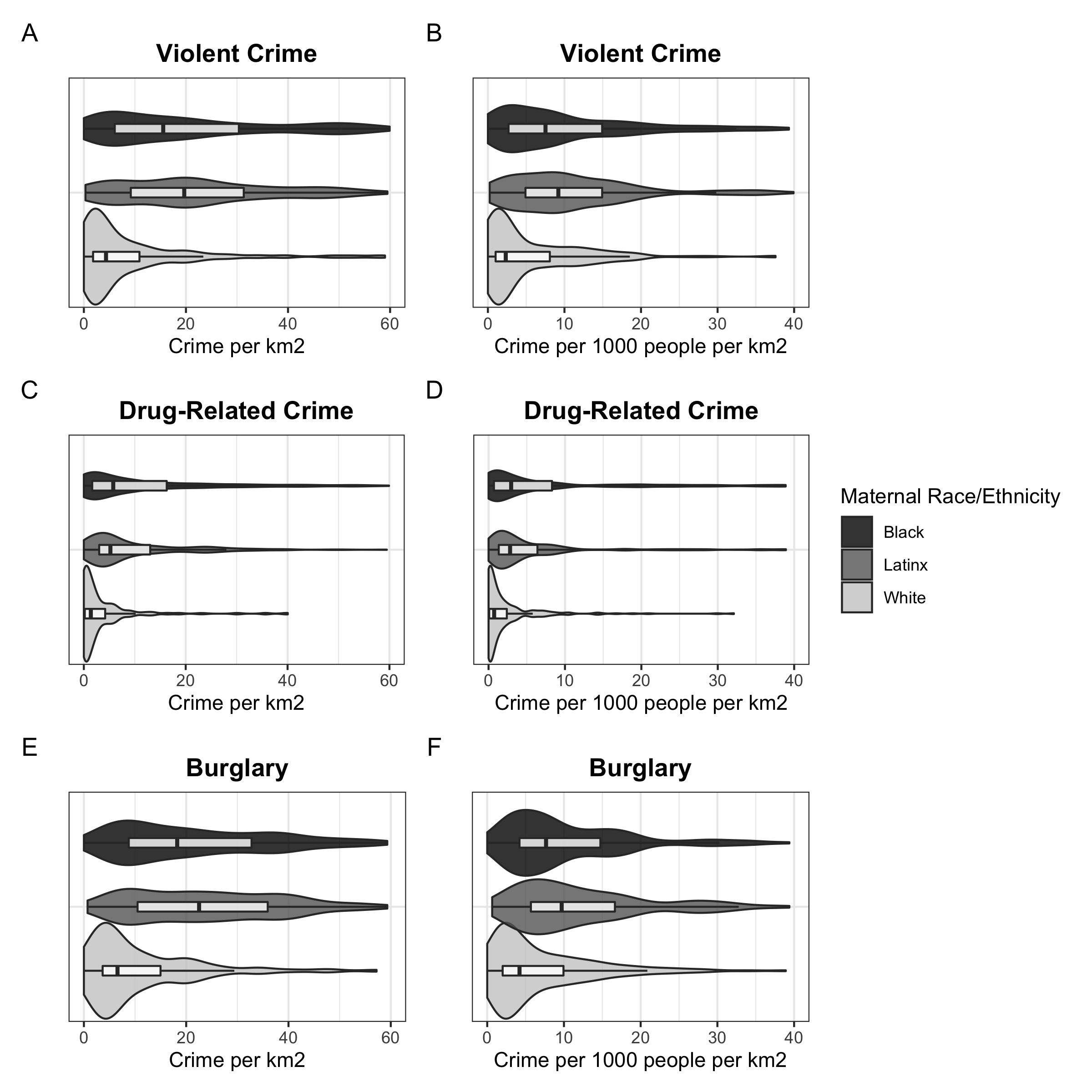
**

**Supplemental Figure 2.** Frequency of (**A**, **B**) violent crime, (**C**, **D**) drug-related crime, and (**E**, **F**) burglary exposure within 400m of participant residence during gestation based on self-reported race and ethnicity. Plots on the left (**A**, **C**, and **E**) display crimes per km^2^, and plots on the right (**B**, **D**, and **F**) display crimes per 1000 people per km^2^. Violin plots display the smoothed kernel density of crime, while boxplots display the 25^th^ quartile, median, 75^th^ quartile, and Tukey whiskers (outliers are not displayed for simplicity).

**
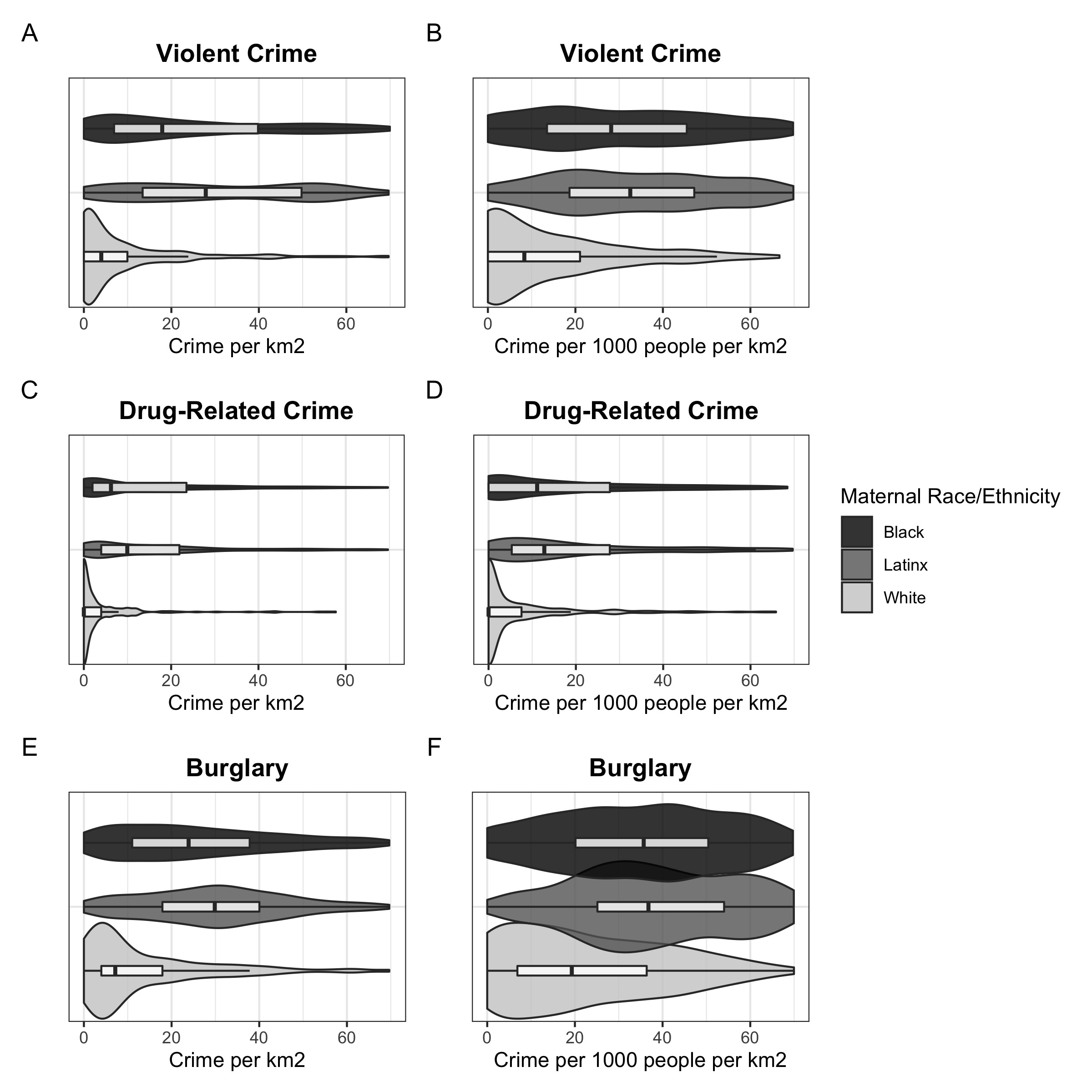
**

**Supplemental Figure 3.** Frequency of (**A**, **B**) violent crime, (**C**, **D**) drug-related crime, and (**E**, **F**) burglary exposure within 1600m of participant residence during gestation based on self-reported race and ethnicity. Plots on the left (**A**, **C**, and **E**) display crimes per km^2^, and plots on the right (**B**, **D**, and **F**) display crimes per 1000 people per km^2^. Violin plots display the smoothed kernel density of crime, while boxplots display the 25^th^ quartile, median, 75^th^ quartile, and Tukey whiskers (outliers are not displayed for simplicity).

**
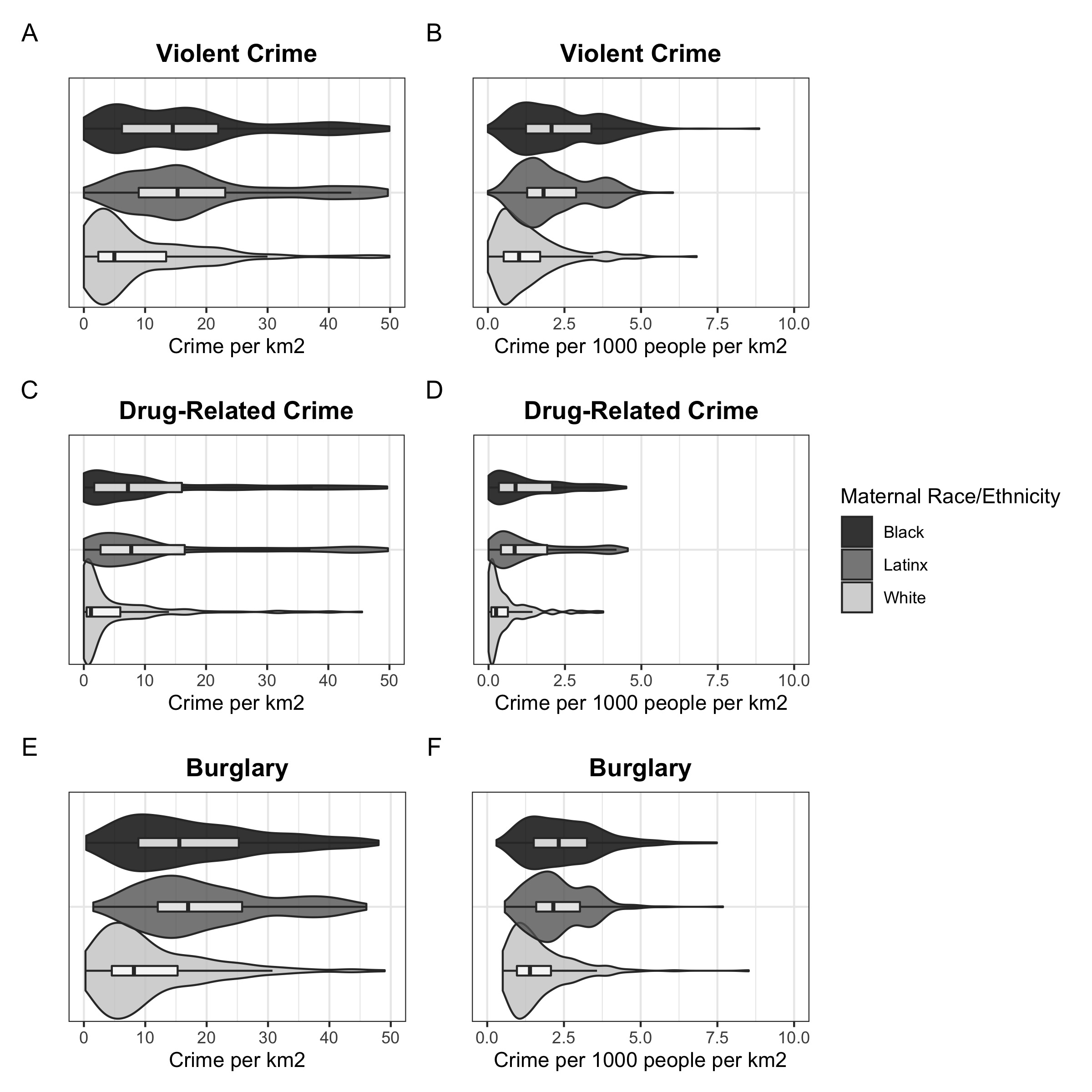
**
